# Influence of different post-interventional maintenance concepts on periodontal outcomes: an evaluation of three systematic reviews

**DOI:** 10.1186/s12903-016-0244-6

**Published:** 2016-07-18

**Authors:** Stefanie J. Gartenmann, Iris Dörig, Philipp Sahrmann, Ulrike Held, Clemens Walter, Patrick R. Schmidlin

**Affiliations:** Clinic of Preventive Dentistry, Periodontology and Cariology, Center of Dental Medicine, University of Zurich, Plattenstrasse 11, CH-8032 Zurich, Switzerland; Horten Centre for Patient-Oriented Research and Knowledge Transfer, Department of Internal Medicine, University of Zurich, Zurich, Switzerland; Department of Periodontology, Endodontology and Cariology University Center for Dental Medicine (UZB), Basel, Switzerland

**Keywords:** Debridement, Flap surgery, Regeneration, Maintenance, Antiseptics

## Abstract

**Background:**

To selectively review the existing literature on post-interventional maintenance protocols in patients with periodontal disease receiving either non-surgical or surgical periodontal treatment.

**Methods:**

Three systematic reviews with different periodontal interventions, i.e. scaling and root planing (SRP), SRP with adjunctive antibiotics or regenerative periodontal surgery were evaluated focusing on their post-interventional maintenance care. Due to the early publication of one review an additional literature search update was undertaken. The search was executed for studies published from January 2001 till March 2015 through an electronic database to ensure the inclusion of resent studies on SRP. Two reviewers guided the study selection and assessed the validity of the three reviews found.

**Results:**

Within the group of scaling and root planing alone there have been nine studies with more than three appointments for maintenance care and five studies with more than two appointments in the first 2 months after the intervention. Chlorhexidine was the most frequently used antiseptic agent used for 2 weeks after non-surgical intervention. Scaling and root planing with adjunctive antibiotics showed a similar number of visits with professional biofilm debridement, whereas regenerative studies displayed more studies with more than three visits in the intervention group. In addition, the use of antiseptics was longer and lasted 4 to 8 weeks after the regenerative intervention. The latter studies also showed more stringent maintenance protocols.

**Conclusions:**

With increased interventional effort there was a greater tendency to increase frequency and duration of the maintenance care program and antiseptic agents.

## Background

Colonization by a pathogenic biofilm is recognized as the primary etiologic factor for the initiation and progression of periodontitis [1]. Despite the fact that host and environmental factors may significantly contribute to the resulting inflammatory process [2], it has been convincingly shown that professional supra- and subgingival biofilm control is able to control disease initiation and progression [3]. The effective control and management of the supra- and subgingival biofilm is traditionally performed by mechanical means, such as hand instruments and/or ultrasonic debridement [4]. Further methods include air-polishing devices with various inserts and powders, the latter preferably being effective in removing biofilms but low-abrasive dental hard tissues [5, 6].

Thorough non-surgical scaling and root planing (SRP) was demonstrated as an important part of successful periodontal treatment, especially on deeper periodontal pockets [7]. The results of such treatment may only be maintained in the long-term when an effective supragingival plaque control is performed, and a regular supportive periodontal treatment (SPT) is applied [1, 8]. In addition, a body of evidence shows the benefit of systemically administered antibiotics as an adjunctive to SRP, particularly in patients with aggressive periodontitis and in those with advanced chronic disease [9, 10].

However, in distinct clinical situations with local defects, e.g. in teeth with furcation involvement or in single-rooted teeth with vertical bone defects, residual increased probing pocket depth (PPD) might persist after non-surgical therapy and require further treatment, e.g. surgical interventions, in order to prevent ongoing loss of attachment and tooth loss [11, 12].

Different studies have analyzed the effects of supervised maintenance care after periodontal therapy eg. subgingival scaling and root planing or surgical intervention. Such maintenance programs included the adjunctive use of antiseptic rinsing followed by professional supragingival cleanings [13, 14]. These supervised maintenance care recommendations are mostly given after elaborate, regenerative periodontal surgery.

However, there is no comparative study or systematic review available, which evaluates the influence of different approaches on clinical outcomes. Therefore, the purpose of this study was to assess post-interventional maintenance protocols in terms of frequency and adjunctive antiseptic infection control for three different treatment modalities concerning infectious periodontal conditions: after non-surgical periodontal therapy with and without systemic antibiotics and regenerative surgical interventions.

The following specific questions were addressed as follows:

1. In a patient population with chronic periodontal disease or periodontal disease with infrabony defects, who underwent different periodontal interventions, which frequency of post interventional maintenance was applied?
2. Is there a difference in pocket depth reduction among the same groups of periodontal therapy with different recall maintenance protocols?

## Methods

### Protocol

The present article merged and screened three already existing systematic reviews, that assessed three different treatment options: SRP [15], systemic antibiotics (amoxicillin and metronidazole) as adjunctive to SRP [16] and regenerative periodontal surgery [17]. All three reviews covered different periodontal therapeutic procedures. The intention of this article was not to compare these primary therapeutic concepts but to expose the measures that were taken after each of these therapies, to put a specific light on the post- interventional protocols and to elaborate any potential differences between the different therapeutic approaches. All studies within the reviews did not show any overlap in the articles the authors have chosen.

Two [16, 17] of the three reviews were fitted to match the current PRISMA (Referred Reporting Items for Systematic Review and Meta-analyses) criteria for reviews [18]. An older systematic review did not follow up to date protocol recommendations [15] due to its earlier publishing date. To assure up-to-dateness and to avoid missing current articles a new literature search was undertaken as described below:

### Eligibility criteria for additional search

Following parameters of the publications needed to be presented in order to be eligible for inclusion:

(1) The articles needed to be randomized controlled trials (RCT) or controlled clinical trials on periodontal treatment with a follow-up of at least 12 months or more, written in English. (2) Patients with chronic periodontitis by the age of at least 20 years. (3) A recording of maintenance care plan of at least 2 months post intervention.

### Outcome measures

The main focus of this study was to filter out different maintenance strategies after any periodontal intervention such as frequency of appointments after SRP and periodontal surgery. In addition changes in periodontal probing depth (PPD) were extracted as primary parameter outcome for meta-analysis. Secondary parameter outcome such as recession (REC), clinical attachment level (CAL) or plaque index (PI) were not part of this meta-analysis due to the non-homogenous data presentation in the single studies. Since the data of probing depth at the requested time points were missing in the non-surgical interventions, only guided tissue regeneration (GTR) studies, which adequately reported on this parameter could be included in the forest-plot.

### Additional analysis and information sources

Due to one study’s early publishing date, the literature search was updated and the electronic databases MEDLINE and Cochrane (Oral Health Group Specialist Trials Register) were consulted again for studies published from January 2001 to March 2015, while the search strategy was re-formulated based on the suggested three complexes “non-surgical therapy” AND “surgical therapy” AND types of studies. Two independent reviewers (ID and PRS) screened for additional titles written in English and searched for possible inclusion criteria, which would match this study’s review protocol. The following modified MeSH terms were used according to the original publication [15]:

- *Disease:*
  “periodontics” OR “periodontal disease”
- *Intervention:*
  “non-surgical therapy” OR “surgical therapy” OR “dental scaling” OR “root planing” OR “dental-prophylaxis” OR “initial therapy” OR “debridement” OR “nonsurgical” OR “non-surgical” OR “periodo*” OR “gingivectomy” OR “periodontal pocket surgery” OR “surgical flaps” OR “modified Widman flap” OR “access” OR “Kirkland” OR “osseous surgery” OR “apically repositioned” OR “coronally”
- *Study design:*
  “longitudinal studies” OR “comparative study” OR “clinical trial”

### Influence of maintenance on therapy

In order to assess the influence of different maintenance protocols, the probing depth reduction served as a clinical outcome. The data on mean and standard deviation of probing depth reduction were extracted from each of the included studies for meta-analysis. Because of differences in the observation period across studies, only those studies were pooled that had somewhat similar follow-up frequencies. Due to a large amount of heterogeneity between studies (I^2^ > 50 %), a random effects model was necessary for pooling. All analyses were performed with R [19]. The studies were arranged in the following categories: protocol 1 (two or less recall visits within the 2 months) and protocol 2 (three or more visits within the 2 months after periodontal intervention). The duration of the use of antiseptic agents were categorized into A, B and C. CHX/A accounts for the use of antiseptics lasting for 2 weeks of rinsing after periodontal intervention. CHX/B required rinsing of 4 weeks and CHX/C of up to 8 weeks after periodontal intervention. For example protocol 2 and CHX/C displays the most vigorous maintenance protocol compared to protocol 1 and CHX/A being the least vigorous post interventional maintenance care (Fig. 2).

### Quality assessment

Studies within the three systematic reviews were methodologically screened by two reviewers in order to assess the quality and a potential risk of bias [15–17].

### Summary of measures

Throughout the three systematic reviews there have been a variety of different maintenance protocols. All studies, which utilized an antiseptic agent, included chlorhexidine (CHX) into their maintenance program, whereas the concentration and duration varied among the studies. If mentioned, all studies provided supragingival cleanings, in two cases oral hygiene instructions and motivation was given. The frequency of follow-up intervals throughout the different reviews was heterogenic.

### Evaluation of the maintenance programs

The following aspects of the post-interventional maintenance protocols were analyzed. The recall frequency including mechanical re-instrumentation and/or re-motivation in the first 2 months after the intervention was arbitrarily identified, whether or not antiseptic rinsing was utilized (active ingredient, concentration, frequency and duration). The results were organized in a subgroup analysis assessing the change of pocket depth reduction. Subgroups were defined as following: (I) The recall frequency during the first 2 months with 1 = two or less visits (≤2) and 2 = three or more visits within the 2 months (≥3) and (II) the duration of adjunctive use of antiseptics for (A) two or less than 2 weeks, (B) Up to 4 weeks and (C) more than 5 weeks. According to this classification the lowest level of maintenance strategy was therefore 1A and the highest-level 2C. Based on this classification system design, further subgroup combinations were possible.

## Results

### Study selection

**Fig. 1:**
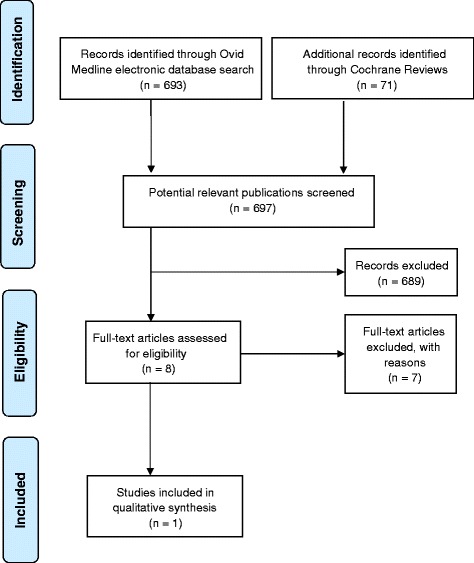
Additional search for non-surgical therapy based on the protocol by Heitz-Mayfield and co-workers (2002) [15]

In total, three reviews were identified by the electronical database search. Since the date of the publication by Heitz-Mayfield and co-workers dating back to 2002 was not up-to-date, an additional investigation was initiated. The latter revealed another 697 publications. After the independent screening procedure by two of the authors (I.D. und P.R.S.), eight studies were included for the full-text analysis. Finally, one additional study met the inclusion criteria and was entered in Table 1 for analysis [20] (Fig. 1).

**Table 1:** List of the analyzed studies dealing with the non-surgical approach [15] (updated 2015)

Postoperative controls	Antiseptics Application				A: 1 + 2 weeks B: 3 + 4 weeks C: 5+ weeks		Maintenance protocol in the first 2 months	1: ≤ 2x 2: ≥ 3x
Study	Antiseptic	Galenic formulation	Application frequency	Duration	Category	Supragingival cleaning	Intervals (weeks)	Category
Pihlstrom & Ortiz 1981	n.r.					yes	Recall frequency varied, 3–4x/yr	1
Lindhe & Westfeldt 1982	CHX	0.2 %	2x/d	2 weeks	A	yes	6 month every 2nd week, then 3 month rec for 18 month	2
Pihlstrom & McHugh 1983	n.r.					yes	Recall 3–4x/yr	1
Lindhe & Westfeldt 1984						yes	6 month every 2nd week, then 3 month rec for 18 month	2
Pihlstrom & Oliphant 1984	n.r.					yes	Recall frequency varied, 3–4x/yr	1
Isidor & Karring 1984	CHX	0.2 %	2x/d	2 weeks	A	yes	2 year 1, professional prophylaxis 3-monthly year 2, 6-monthly years 3, 4 & 5 – subgingival debridement	2
Lindhe & Nyman 1985	CHX	0.2 %	2x/d	2 weeks	A	yes	every 2nd week for 12 weeks, 3 month recall	2
Kaldahl & Kalkwarf 1996	n.r.					yes	3 month intervals	1
Isidor & Karring 1986	CHX	0.2 %	2x/d	2 weeks	A	yes	2 year 1, professional prophylaxis 3-monthly year 2, 6-monthly years 3, 4 & 5 – subgingival debridement	2
Ramfjord & Caffesse 1987	n.r.					yes	1,2,3,4, then every 3 months	2
Kaldahl et al. 1988	n.r.					yes	2,4,7 (subgingival plaque removal)	2
Kalkwarf et al1988	n.r.					yes	1,2,4,7, then 3 month recall	2
Kalkwarf et al. 1989	n.r.					yes	1,2,4,7, then 3 month recall	2
Serino et al. 2001	CHX	0.2 %	2x/d	2 months	C	yes	3–4x/year, re-examinations after 1, 3, 5, 13 years	1

### Description of study maintenance protocol

The analysis of the maintenance protocols included reviews with three different periodontal approaches. Mayfield et al. (2002) evaluated studies dealing with a non-surgical approach whereas Zandbergen and co-workers in 2013 analyzed studies treating periodontal disease with conventional non-surgical therapy and the adjunctive use of systemically administered amoxicillin and metronidazole. Further Graziani et al. (2012) focused on studies dealing with regenerative therapy. Taking into account the heterogenic study designs of 78 studies in total depending on the treatment, most of the studies listed in the reviews followed a specific maintenance protocol after treatment. A detailed overview of the different maintenance protocols is given in Tables 1, 2, and 3. This analysis’s keynote was type and concentration of antiseptic formula as well as instruction given concerning intake frequency and duration. In addition, the type of maintenance and intervals after treatment were defined. With regard to the antiseptic rinsing, all studies used chlorhexidine (CHX) in concentrations ranging from 0.06 to 0.2 %. The latter and therefore highest concentration was used in roughly 50 % of the studies. Patients were advised to rinse twice daily in most articles, whereas the individual antiseptic rinsing period varied significantly. The minimum concentrations and time for adjunctive chemical plaque control was found in the SRP with adjunctive use of antibiotics group of one study. There, patients were advised to use 0.06 % antiseptic chlorhexidine once daily for 8 days [21–23] (Table 2). The most extensive antiseptic regimen was revealed in studies with regenerative treatment. The minimum rinsing concentrations of 0.12 % chlorhexidine and a rinsing period of up to 11 weeks going up to a concentration of 0.2 % and a rinsing duration of 10 weeks could be observed [24, 25] (Table 3). The recall duration and frequency including professional plaque control also varied throughout all the included studies as listed in the reviews. Table 4 compares the antiseptic duration and recall frequency 2 months after treatment within the different treatment groups. The non-surgical approach group chose to administer the higher chlorhexidine concentration with 0,2 %. However, only five out of 14 studies in the review by Mayfield et al. 2002 advised adjunctive antiseptic rinsing, whereas all 14 studies displayed post-interventional recall intervals of at least once every 2 months. Nine studies exhibited a recall interval of equal three if not more visits. Not listed in Table 4 are the five studies within the scaling and root planing group who merely appeared 3 months after the intervention. Whether or not patients were advised to rinse with antiseptic agents is not mention in the studies (Table 1). Seventeen out of 34 studies in the group with SRP and the use of adjunctive antibiotics listed in the review by Zandbergen and co-workers (2013) prescribed chlorhexidine mouth rinse. In this review with 34 studies in total 20 studies performed a post-interventional maintenance protocol of less than two visits. Two studies performed more than three visits within the past 2 months following the procedure. The regenerative approach showed 27 out of 30 studies with adjunctive use of antiseptic agents and 26 studies included a maintenance protocol within the 2 months. 23 studies scheduled their patients more than three times in the 2 months following the regenerative intervention. Reason for this strict maintenance protocol as described by the authors was to remove sutures after surgery, polishing for plaque control due to its tooth brushing abstention at the site of surgery and finally regular supragingival cleanings [17]. Overall, the more sophisticated a treatment intervention was the greater the tendency to increase maintenance frequency, mouth-rinse concentration and duration.

**Table 2:** Detailed list of analyzed studies dealing with non-surgical periodontal therapy with adjunctive use of amoxicillin and metronidazole [16]

Postoperative controls		Antiseptics Application				A: 1 + 2 weeks B: 3 + 4 weeks C: 5+ weeks	Maintenance protocol in the first 2 months	1: ≤ 2x 2: ≥ 3x
Study	Antiseptic	Galenic formulation	Application frequency	Duration	Category	Supragingival cleaning	Intervals (weeks)	Category
Van Winkelhoff et al. 1992	n.r.					n.r.	clinical measurements at 3 to 9 months (4.9 ± 2 months)	
Winkel et al. 1998	n.r.					hygiene instructions	week 21	1
Müller et al. 1998	CHX	0.10 %	n. r.	2 weeks	A	n.r.	clinical measurements at 3 months	
Flemmig et al. 1998	CHX	0.06 %	1x/d	8 days	A	yes	10 days, 3, 6, 9 and 12 months	1
Berglundh et al. 1998	n.r.					n.r.	clinical parameters at 2 and 12 months	
Winkel et al. 2001	n.r.					yes	week 6	1
Ehmke et al. 2003	CHX	0.06 %	1x/d	8 days	A	yes	3, 6, 9, 12, 18, 24 months	1
Guerrero et al. 2005	CHX	0.2 %	2x/d	2 weeks	A	yes	2 and 6 months	1
Mombelli et al. 2005	n.r.					yes	10 days, 2, 6 and 12 months	1
Ehmke et al. 2005	CHX	0.06 %	1x/d	8 days	A	yes	3, 6, 9, 12, 18, 24 months	1
Xajigeorgiou et al. 2006	n.r.					yes	week 6	1
Giannopoulou et al. 2006	n.r.					n.r.	clinical parameters at 10 days, 2, 6 and 12 months	
Moeintaghavi et al. 2007	n.r.					n.r.		
Kaner et al. 2007	CHX	0.2 %	2x/d	2 weeks	A	yes	3 and 6 months	1
Moreira et al. 2007	CHX	0.12 %	2x/d	2 months	C	yes	3 month	1
Guerrero et al. 2007	CHX	0.12 %	2x/d	2 weeks	A	yes	2 months	1
Machtei et al. 2008	n.r.					oral hygiene motivation	every 2nd week for 3 months	2
Johnson et al. 2008	CHX	0.12 %	2x/d	30 days	C	n.r.	clinical parameters at 3 and 6 months	
Akincibay et al. 2008	n.r.					n.r.	clinical measurements at 30, 60, 90 days	
Valenza et al. 2009	n.r.					yes	2, 6 and 12 months	1
Del Peloso et al. 2009	n.r.					yes	every month for 6 months	1
Cionca et al. 2009	CHX	0.2 %	2x/d	10 days	A	yes	1 week, 3 and 6 months	1
Yek et al. 2010	n.r.					yes	monthly up to 6 months	1
Cionca et al. 2010	CHX	0.2 %	2x/d	10 days	A	yes	1 week, 3 and 6 months	1
Mestnik et al. 2010	CHX	0.12 %	2x/d	60 days	C	n.r.	clinical measurements at 3 months	
Heller et al. 2011	CHX	0.12 %	2x/d	45 days	C	yes	3 months	1
Rodrigues et al. 2011	n.r.					yes	1, 3 and 6 months	1
Silva et al. 2011	n.r.					n.r.	clinical measurements at 3 months	
Varela et al. 2011	CHX	0.12 %	2x/d	45 days	C	yes	3 months	1
Baltacioglu et al. 2011	n.r.					n.r.	clinical measurements at 2 months	
Aimetti et al. 2012	CHX	0.2 %	2x/d	2 months	C	yes	every 2nd week for 6 weeks, then every 2 months up to 6 months	2
Casarin et al. 2012	n.r.					n.r.	clinical measurements at 3 and 6 months	
Goodson et al. 2012	CHX	0.12 %	2x/d	2 weeks	A	yes	3, 6, 12, 18, 24 months	2
Oliveira et al. 2012	CHX	0.12 %	2x/d	60 days	C	n.r.	clinical measurements at 6 months

**Table 3:** Detailed list of analyzed studies dealing with regenerative periodontal therapy, i.e. GTR [17]

Postoperative controls			Antiseptics Application			A: 1 + 2 weeks B: 3 + 4 weeks C: 5+ weeks	Maintenance protocol in the first two months	1: ≤ 2x 2: ≥ 3x
Study	Antiseptic	Galenic formulation	Application frequency	Duration	Category	Supragingival cleaning	Intervals (weeks)	Category
Yukna et al. 1985	n.r.	n.r.	n.r.	n.r.		yes	at 10, 20 and 30 days and 3, 6, 9 and 12 months	2
Cortellini et al. 1995a	CHX	0.2 %	2x/d	3 weeks	B	yes	1,2,3,4,5,6 (and after membran removal 1,2,3,4)	2
Mora & Ouhayoun 1995	CHX	0.2 %	n.r.	10 days	A	yes	every 10 days for the 1st month, 2x a month for months 2 and 3 and then every 3 months for 9 months	2
Cortellini et al. 1996	CHX	0.2 %	2x/d	3 weeks in the MWF group 2 months bioresorbable gr 6 weeks in the ePTFE gr		yes	1,2,3,4,5,6, then every month for 12 months	2
Tonetti et al. 1996	CHX	0.2 %	2x/d	3 weeks	B	yes	weekly for 6 weeks. After membran removal weekly for 4 weeks, then monthly up to 12 months	2
Mora et al. 1996	CHX	0.2 %	2x/d	4 weeks	B	yes	weekly 4 weeks, professional maintenance regimen after membran removal until 12 month	2
Zamet et al. 1997	CHX	0.2 %	2x/d	4 weeks	B	yes	1,2,3,4, then every 4 weeks up to 3 months and then every 3 months up to 1 yr	2
Heijl et al. 1997	CHX	0.2 %	n.r.	4–6 weeks	C	n.r.	n.r.	
Mayfield et al. 1998	CHX	0.2 %	2x/d	6 weeks (test) 3 w (controlgr.)	B	yes	weekly for 4 weeks, then monthly until 6 month, then every 4–6 weeks up to 1 yr	2
Okuda et al. 2000	CHX	0.12 %	3x/day	6 weeks	C	yes	weekly for 6 weeks, then monthly up to 12 months	2
Zybutz et al. 2000	CHX	0.12 %	2x/d	8 weeks	C	yes	2,4,6,8,12 weeks	2
Ratka-Kruger et al. 2000	CHX	0.2 %	3x/day	6 weeks	C	yes	at 3, 6 and 12 months	1
Sculean et al. 2001	CHX	0.2 %	2x/d	6 weeks	C	yes	every 2nd week for 2 months, then monthly up to 12 months	2
Zucchelli et al. 2002	CHX	0.12 %	2x/d	11 weeks	C	yes	Weekly for 11 weeks, then monthly up to 1 yr	2
Tonetti et al. 2002	CHX	0.12 %	2x/d	4 weeks	B	yes	at weeks 1, 2, 3, 4, 6. Then 3,6,9 month recall	2
Wachtel et al. 2003	CHX	0.2 %	2x/d	4 weeks	B	n.r.	n.r.	
Sculean et al. 2003	CHX	0.2 %	2x/d	4 weeks	B	yes	every 2nd week for 2 months,then monthly up to 12 months	2
Sculean et al. 2004	CHX	0.2 %	2x/d	6 weeks	C	yes	every 2nd week for 2 months,then monthly for the 1st yr, after 1st yr for 5 yrs every 3 months	2
Tonetti et al. 2004	CHX	0.12 %	2x/d	4 weeks	B	yes	at weeks 1, 2, 3, 4, 6 and 8. Then at 3, 6 and 9 months	2
Vouros et al. 2004	CHX	0.12 %	2x/d	4 weeks	B	yes	monthly up to 12 months	1
Francetti et al. 2004	CHX	0.12 %	2x/d	6 weeks	C	yes	recall monthly during 1 yr, then at 18 and 24 month	1
Francetti et al. 2005	CHX	0.12 %	2x/d	6 weeks	C	n.r.	n.r.	
Aimetti et al. 2005	CHX	0.2 %	2x/d	3 weeks	B	yes	1,2,3,4, then every month for 12 months	2
Sculean et al. 2005	CHX	0.2 %	2x/d	4 weeks	B	yes	every 2nd week for 2 months,then monthly up to 12 months	2
Sculean et al. 2007	CHX	0.2 %	2x/d	4 weeks	B	yes	every 2nd week for 2 months,then monthly up to 12 months after 1st yr for 5 yrs every 3 months	2
Sculean et al. 2008	CHX	0.2 %	2x/d	6 weeks	C	yes	every 2nd week for 2 months,then once per month for 1 yr, then every 3 months for 10 yrs	2
Kasaj et al. 2008	CHX	0.2 %	2x/d	4 weeks	B	yes	every 2 week for 2 months, after every 4 weeks	2
Fickl et al. 2009	n.r.					n.r.		
Stein et al. 2009	CHX	0.2 %	2x/d	4 weeks	B	yes	every 2nd week for 2 months,then monthly up to 12 months	2
Cortellini et al. 2011	CHX	0.12 %	2x/d	4 weeks	C	yes	1,2,3,4,5,6, then 3 month recall for 1 yr	2

**Table 4:** Comparisons of the different maintenance programs according to the use of antiseptic rinsing (CHX 0,06-0,2 %) and recall frequency (number of visits)

	Duration of the use of antiseptics CHX			Number of recalls within the first 2 months Protocol	
	First 2 weeks (A)	Up to 4 weeks (B)	Up to 8 weeks (C)	≤2 visits (1)	≥3 visits (2)
Non-surgical only (*n* = 14)	4	-	1	5	9
non-surgical plus systemic antibiotics (*n* = 34)	10	-	7	20	2
Regenerative surgical procedures (*n* = 30)	1	15	11	3	23

Duration of antiseptic use CHX: A = two or less than 2 weeks; B = up to 4 weeks; C = more than 5 weeks. Number of recall visits following periodontal treatment within the first 2 months; Protocol: 1 = two or less visits; 2 = three or more recall appointments within the first 2 months

### Influence of different maintenance programs on probing depth reduction

**Fig. 2:**
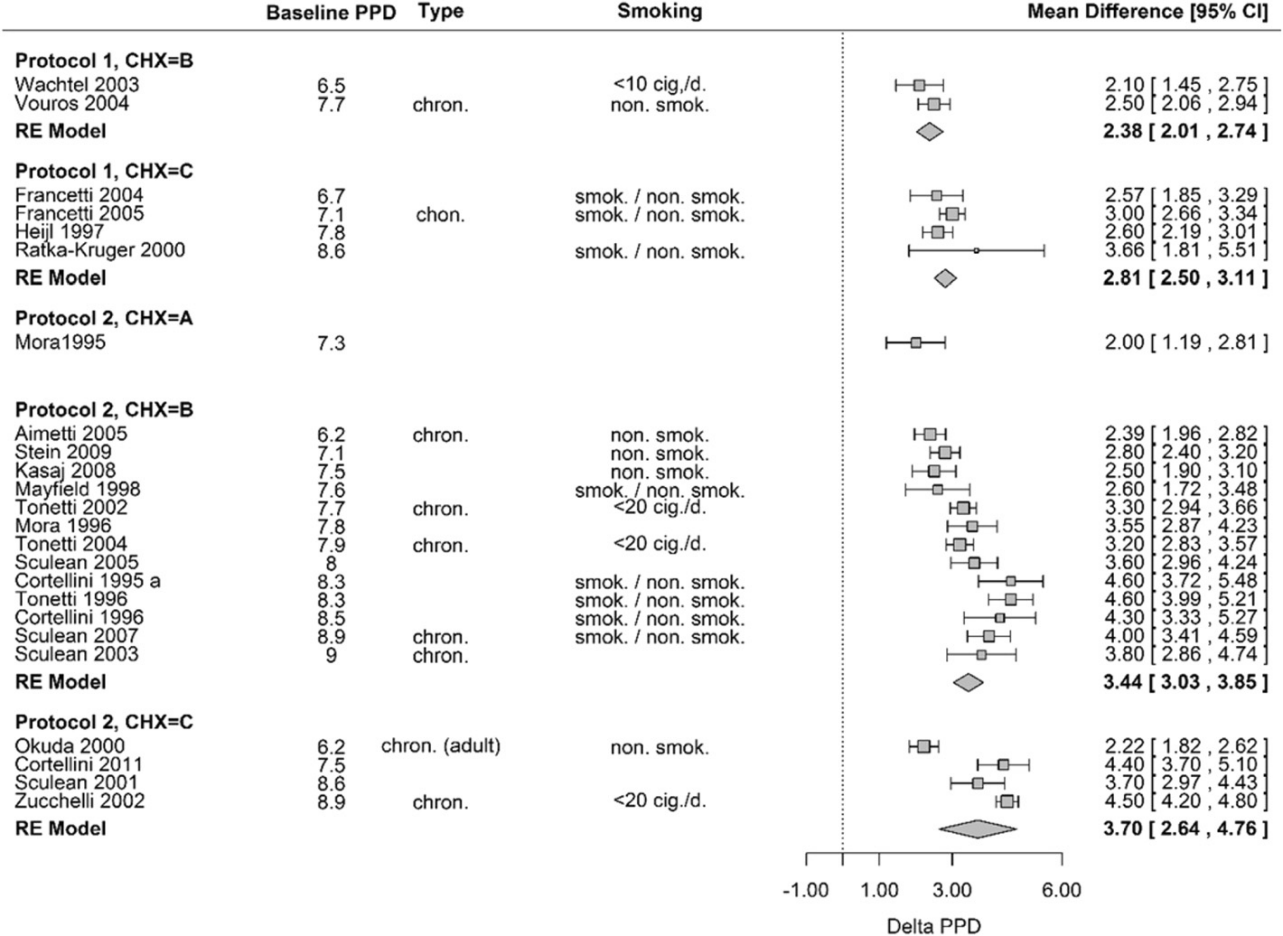
Forest plot of the studies on regenerative therapy included in the meta-analysis [17]

A graphical representation of the results is given in a forest plot (Fig. 2). However, to start with, it is important to highlight the nature of this analysis. It is purely based on the data of the regenerative procedure presented by the systematic review from Graziani and co-workers (2012). There have been complications along the way to extract the needed data from the other reviews. Therefore, these pooled results cannot be statistically analyzed, directly compared and interpreted.

Nevertheless, the regenerative studies with more recall interventions and longer duration of antiseptic agents after surgery displayed a greater PPD reduction compared to the studies with lower protocol interventions and antiseptic agents i.e. longer rinsing periods and/or more recall visits looking at the pooled data. The highest mean difference was observed in protocol 2/CHX = C with a mean probing pocket depth difference of 3.7 mm. In general, there has been an increase of the observed effect with increasing baseline PPD. Protocol 1/CHX = C and protocol 2/CHX = C – both groups having four studies with a comparable range of baseline probing pocket depths (PPD) – the mean differences greatly differed and accounted for 2.81 mm versus 3.70 mm, respectively. Both groups show different probing depth reductions with different protocols and rinsing durations due to its type of surgical intervention and patient care needs. Again the data presented and its evaluation was extracted from the forest plot done only on the studies on regenerative therapy.

### Quality assessment

Graziani et al. and Zandbergen et al. demonstrated a quality assessment to estimate the risk of bias. Nine articles were using adequate methods of study design, unclear methods were used in 21 articles and inadequate methods in eight articles [17]. Out of 28 studies, 15 studies demonstrated low potential risk of bias. The remaining studies showed moderate to high risk of bias [16]. Heitz-Mayfield et al. justified missing quality assessment with a limited number of studies [15].

## Discussion

Based on the premises of peer-reviewed papers, this study approach was to pool the evidence and extract the data regarding the maintenance care intervals and procedures. The aim of this article was to put a specific light on the post-interventional protocols and to elaborate any potential differences between the different therapeutic approaches, but not to compare the actual outcomes due to obvious reasons. The performed summary must not be understood as an inadmissible comparison of the clinical results from different treatment approaches. Nevertheless, the latter appraised differences of post-interventional maintenance programs and their impact on periodontal healing was done for the surgical interventions in regard to pocket depth reduction.

As an interesting main finding, the studies analyzed in the three reviews, showed different post-interventional plaque control strategies with regard to chemical and mechanical plaque control regimens among the different treatment groups. For instance, 1 to 2 weeks reflects a reasonable time span after surgical therapy until sutures are removed. Other time points were adjusted to 1 and 2 months. The regenerative surgical approach showed the highest degree of maintenance efforts after the intervention. An explanation for the continuous monitoring is the nature of regenerative therapy since this therapy is invasive and expensive. An explanation for the continuous monitoring is the nature of regenerative therapy since this therapy is invasive and expensive. Nevertheless, a prospective clinical study on patient undergoing one-stage full-mouth scaling and root planing has demonstrated a statistical significant benefit in probing depth and clinical attachment gain after 3 months of extensive use of CHX mouth rinse. [26].

Due to the fact that all studies included in this study were part of peer-reviewed reviews, the evaluation of outcome measurements such as PPD and CAL was not being weighed against each other, only the mean PPD difference on the regenerative studies presented in the forest plot. However, one systematic review did not meet the current standard requirements for systematic reviews due to an earlier publishing date. Hence, a new search was undertaken to compensate for the lack of up-to-dateness. In addition, the classification to evaluate the maintenance protocol was arbitrarily set, which might be considered a shortcoming of the present study. However, it reflects potentially relevant time frames in the course of periodontal therapy.

Postoperative success is determined by many factors, such as anatomical and technical factors, patient compliance, plaque control and cigarette smoking. All these are factors that can directly affect the predictability of periodontal regeneration [27]. Thus, low plaque scores have shown to reduce the risk of membrane exposure, infection and guarantee better complication management [25, 28]. These factors inevitably also lead to more stringent protocols, which is mainly justified by infection control and healing optimization. Common procedure such as the intake of adjunctive antibiotics or anti-inflammatory medication during regeneration could also be one factor for a favorable outcome.

The importance of postoperative plaque control in determining the outcome of periodontal surgery is well established and recognized in the literature for a long time [29].

In contrast, studies using systemic antibiotics as an adjunct to SRP disclosed an opposite tendency. Almost no chemical plaque control was done and patients were left unsupervised until reevaluation after 3 months. Non-surgical therapy with systemic antibiotics is considered to be a more cost-effective treatment alternative in contrast to sophisticated regenerative surgery. Its aim is to reduce the need for any surgical therapy [30, 31]. In addition, less postoperative complications may be expected due to the fact that neither surgery has been performed nor foreign materials have been implanted. Quite to the contrary, patients were only under the guard of antibiotics. Overall, two different periodontal procedures with their specific therapy goals, extend of treatment site as well as different healing needs make it challenging to compare and evaluate the results. However, plaque scores after 3 months were quite high in some studies and reached a plaque index of above 30 % at reevaluation [32–34]. Some studies did not even report on plaque indices, which made a more detailed assessment of this important data impossible. Therefore, it remains unclear to what extent decreased plaque levels would have led to a better clinical outcome. In contrast, evidence suggests that the occurrence of re-established plaque may lead to recolonization, less healing and persistence of the original pathology [35, 36].

Missing quantitative data on probing depth reductions at the requested time points also made it impossible to assess and compare the results of the non-surgical interventions and include the data in a forest-plot. Therefore, only adequately reported GTR studies could be included into this analysis.

Periodontal sites, which could be influenced, were suprabony and infrabony defects as well as pockets with furcation involvement. Inarguably, the role and potential of adequate plaque control during therapy and afterwards have an impact on the subgingival microbiota [37]. The importance of an adequate maintenance protocol for the success or failure in periodontal therapy has therefore been introduced as an achievable goal for decades [38].

## Conclusion

By tendency, regenerative studies showed a longer duration of antiseptic mouth rinse and higher quantity of maintenance protocol compared to non-surgical approaches. However, sophisticated treatment should not be a causal reason for vigorous recall intervals more an evidence based reason. Till today there is little evidence on how elaborate a post treatment or postoperative protocol should be in order to benefit the patient. Carefully executed prospective studies on this topic are still warranted.

## Abbreviations

CAL, clinical attachment level; GTR, guided tissue regeneration; PI, plaque Index; PPD, probing pocket depth; PRISMA, referred reporting items for systematic review and meta-analyses; RCT, randomized clinical control trail; REC, recession; SPT, supportive periodontal ttreatment; SRP, scaling and root planning
